# Rapid Review: Ascertaining the Type of Potentially Life-Threatening Cardiovascular Events and Eventual Cardiovascular Mortality From Antipsychotic Use in Clinical Practice

**DOI:** 10.1192/bjo.2024.190

**Published:** 2024-08-01

**Authors:** Reagan Lee, Oscar Han, Jingjing Wang

**Affiliations:** 1 Edinburgh Medical School, University of Edinburgh, Edinburgh, United Kingdom.; 2 School of Medicine and Population Health, University of Sheffield, Sheffield, United Kingdom

## Abstract

**Aims:**

According to the World Health Organization (WHO), there has been a 13% increase in mental health and substance abuse disorders within the last decade. Typical and atypical antipsychotics are the most common treatment mechanisms for mental health-related disorders such as schizophrenia, depression with psychotic symptoms and bipolar disorders. However, antipsychotic usage is associated with more than a 50% increase in CVD such as ischaemic heart disease, resulting in cardiovascular-related mortality.

This review aims to investigate the most common type of cardiovascular event causing mortality due to antipsychotic use.

**Methods:**

A systematic search within PubMed and Medline was conducted on 3 October 2023. Selection criteria were limited to English, full-text studies excluding case reports. The time frame selected was up to 3 October 2023. All studies included adults only. Interventions of focus include typical and atypical antipsychotics licensed in the UK. Outcome measures include cardiovascular mortality/events post-antipsychotic prescription.

**Results:**

13 studies were included out of 1088 records. Studies originated from 4 nations with the most studies coming from the USA (n = 7), UK (n = 2), Taiwan (n = 2) and Canada (n = 2).

The most common antipsychotic reported in the records was risperidone (n = 11), followed by haloperidol (n = 9), olanzapine (n = 8) and quetiapine (n = 8).

From data extraction, the most common cardiovascular events leading to death were sudden cardiac death/arrest (n = 6), ventricular arrhythmias (n = 6), myocardial infarction (n = 4), and heart failure (n = 2).

Due to data heterogeneity, discrete outcome measures were extracted from each record. This included outcomes measuring: relative risk between various groups (n = 9), rate of cardiovascular event per 100 patient year (n = 3), and mortality post cardiovascular event (n = 1).

**Conclusion:**

From this study, ventricular arrhythmias and sudden cardiac deaths were the most common cardiovascular events secondary to antipsychotic use leading to mortality. Owing to patient safety and benefits, patients with psychotic illness are unable to go untreated. They are consequently very vulnerable to the cardiovascular side effects of prescribed high-dose antipsychotic drugs. Despite current monitoring guidelines worldwide, cardiovascular-associated mortality in patients on antipsychotics is still elevated. This may indicate the potential inadequacy of current measures for these patients while demonstrating the need for more aggressive cardioprotective interventions and monitoring.

